# Using geophysics to guide the selection of suitable sites for establishing sustainable earthen fishponds in the Niger-Delta region of Nigeria

**DOI:** 10.1016/j.heliyon.2023.e17618

**Published:** 2023-06-24

**Authors:** Efemena D. Emmanuel, Kennedy O. Doro, Ruth E. Iserhien-Emekeme, Edmund A. Atakpo

**Affiliations:** a Department of Physics, Delta State University, Abraka, Nigeria; b Department of Environmental Sciences, University of Toledo, 2801 W. Bancroft Street, Toledo, OH, 43606, USA

**Keywords:** Aquaculture, Clay content, Geophysics, Electrical resistivity, Induced polarization

## Abstract

Water retention in earthen fishponds throughout a fish farming cycle is challenging due to climate-induced water loss via evapotranspiration, seepages, and lowering of the groundwater table. These processes depend on the soil hydrostratigraphic condition and constitute a major challenge for fish farmers in the Niger-Delta region of Nigeria, where seasonal variations cause groundwater levels to fluctuate. This study assesses the use of non-invasive geophysical methods, including electrical resistivity and induced polarization, to guide the selection of sites with appropriate hydrostratigraphic conditions for establishing earthen fishponds. We combined measurements of electrical resistivity and chargeability distributions to assess the subsurface of two earthen fishpond sites at Ugono-Abraka and Agbarha-Otor areas in the Niger-Delta region of Nigeria. Electrical soundings were acquired at ten locations, while two-dimensional electrical resistivity and Induced polarization were acquired across five transects using Schlumberger and dipole-dipole electrode configurations. The field data were inverted using IP2win, and Diprowin software. The geophysical models were combined with lithological data from soil cores to characterize the subsurface stratigraphy, while measured clay contents were used to estimate infiltration coefficients relying on established petrophysical relationships. The delineated subsurface properties at Ugono-Abraka and Agbarha-Otor show higher variations than assumed by practitioners. The complementary results of low resistivity (20–140 Ωm) and high chargeability (10–50 msec) revealed areas with clay-rich sediments. Soil samples confirmed higher clay contents of up to 10% at Ugono-Abraka and low values of 2% at Agbarha-Otor. Estimated infiltration coefficients are lower at the Ugono-Abraka site (1.6 m/day) compared to Agbarha-Otor (8.4 m/day). This implies variable water loss in the earthen fishponds; hence, we recommend characterizing these variations using non-invasive geophysical methods before establishing medium to large-scale earthen fishponds in the area.

## 1 Introduction

Fish farming is considered one of the fastest-growing animal-based food-producing sectors in Nigeria [1,2] as the country is currently ranked the world's largest producer of “African Catfish” by the Food and Agricultural Organization of the United Nations [3,4]. For a developing country, sustaining the fish farms is critical to improving its food security and achieving the United Nation's sustainable development goals No. 1 and 2. Goal No. 1 aims to end poverty in all forms, while goal No. 2 focuses on ending hunger, achieving food security, improving nutrition, and promoting sustainable agriculture [[5], [6], [7]]. A significant proportion of fish farming involves growing fish in ponds, referred to as fish culture [8]. Fish culture production in Nigeria relies significantly on freshwater earthen ponds for holding, rearing, and harvesting fish [9]. This typically requires retaining the water in place for the entire growing cycle, which is difficult for farmers, depending on the seasonal climatic variations and the site's hydrogeological properties. This study seeks to extend the use of non-invasive geophysical techniques for characterizing the subsurface at earthen fishpond sites.

Identifying suitable sites that can retain water throughout the farming cycle is a major challenge for fish farmers who rely on earthen ponds [[10], [11], [12]]. The ease of access to water and naturally waterlogged lands has led to an increasing operation of aquaculture farms within Nigeria's Niger Delta coastal flood plains [13]. However, the relatively high seasonal fluctuation of the water table between the alternating cycles of rainy (wet) and dry seasons limits the availability of suitable sites for sustainable fishponds [14,15]. This study leverages established relationships between geophysical parameters and soil hydrostratigraphic properties [[16], [17], [18]] to delineate the subsurface lithology at earthen fishpond sites. It seeks to extend the use of geophysical methods to aquaculture farming, particularly to improve the selection of sites with suitable hydrostratigraphic conditions for establishing sustainable earthen fishponds.

**Fig. 1:**
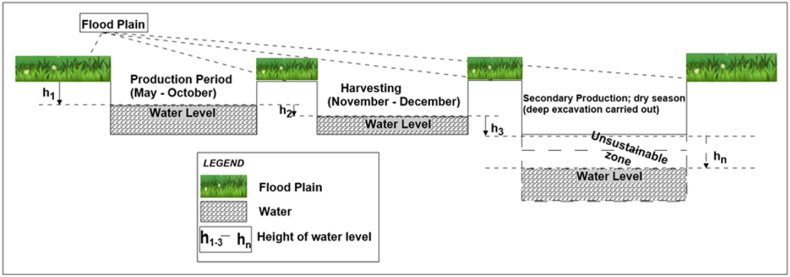
A schematic showing the operational mechanism of an earthen fishpond in the Niger-Delta Region. The ponds from left to right show water level decline with the operation, and lastly, digging to greater depths to assess the water table at greater depth. When water levels are too deep, the ponds are eventually abandoned.

The establishment of fish farms within the Niger-Delta region of Nigeria using earthen fishponds involves digging a selected area to establish a depression that extends through the shallow water table, typically between <1 and 3 m deep, depending on the location (see Fig. 1) [19]. These fishponds are undrainable and groundwater-fed earthen ponds [[20], [21], [22]]. See the Food and Agriculture Organization of the United Nations' training series manual on “Pond construction for freshwater fish culture” for more details on pond types and their properties [[23], [24], [25], [26]]. Although the side of the pond is sometimes lined with polyethylene materials to minimize water loss, its base and sides are sometimes left unlined, which could be areas of water loss via vertical or horizontal seepages [21,27]. The vertical or horizontal flow of water within the subsurface depends on the soil type and its hydraulic properties, such as the infiltration coefficient in the unsaturated zone or hydraulic conductivity in the saturated zone [26,28]. When vertical and/or horizontal flow occurs, water loss results in the fishponds becoming unsustainable. Other potential sources of water loss include evapotranspiration which is also significantly high during a typical dry season with average temperatures of 33° Celsius in Southern Nigeria [[29], [30], [31]]. In critical cases of lowered groundwater table and water loss, the ponds dry up and result in the fish's death, which constitutes a significant financial loss for the farmers. To prevent this, the fish farmers temporarily remove the fish and extend the depth of the ponds by digging deeper (Fig. 1). When this is unsuccessful due to further lowering of the groundwater table, the farmers are forced to sell off the fish and abandon the ponds. Besides the financial losses for farmers, this also constitutes environmental degradation as abandoned fishpond areas are rarely re-filled [27,30]. To address this challenge, it is pertinent to assess potential sites' subsurface stratigraphic and hydrogeological conditions before establishing medium to large-scale fishponds to ensure that they would be capable of holding water year-round or would need to be engineered for such purpose [27]. Integrating such data-informed approaches into local fish farming in Nigeria would improve sustainable agriculture [32].

Understanding the subsurface conditions, including the hydraulic properties of fishpond sites, mostly in tropical areas, has received limited attention [27]. With the lack of documented scientific data, mostly in the Niger Delta region of Nigeria [32,33], site assessments before establishing earthen fishponds rely on indigenous knowledge via visual inspection and judgment of the farmers based on their experience within a given locality. This, however, is associated with high uncertainty exacerbated by climatic and geologic variabilities. Soil cores and test pits are sometimes used to assess the nature of the subsurface sediments, while surrounding wells or shallow hand-dug wells close to the site are used to assess the water table [21]. While these give direct information on the lithological and hydrogeological conditions, they are limited in capturing spatial variations in the subsurface hydrostratigraphic conditions, which is an unresolved challenge in the sustainable use of the earth's shallow subsurface [34]. Like other related challenges requiring detailed knowledge of the subsurface stratigraphic and hydraulic properties, the use of geophysical techniques, either independently or in combination with soil cores and *in situ* hydrogeological testing, significantly improves understanding of the subsurface stratigraphic heterogeneity and estimation of its hydraulic properties such as infiltration coefficient or hydraulic conductivity [16,35].

The use of geophysical methods including electrical resistivity and induced polarization for delineating subsurface hydrostratigraphic heterogeneity has been validated in several studies [36,37]. Also, petrophysical relationships relating measured electrical resistivity to soil hydrogeological properties have been demonstrated [38,39]. However, there is little or no application of this state-of-the-art in geophysics to establishing sustainable aquaculture (earthen fishpond) sites.

The objective of this study was to innovatively use standard geophysical methods i.e., electrical resistivity and induced polarization in combination with soil cores, to assess sites' suitability for establishing earthen fishponds. We showed the use of existing petrophysical relationships for predicting infiltration coefficients of the soil at each earthen fishpond site and assessing potential water seepages within them. We hypothesize that areas with high clay contents will correlate with low resistivity and high chargeability, have low infiltration rates, and retain water longer. Hence, they would be more suitable for establishing a fishpond. This study uses electrical resistivity and chargeability models of the study sites along with soil cores and petrophysical estimates of infiltration coefficient to characterize the soils and their potential to retain water. We, therefore, demonstrate the potential of using geophysics to address challenges beyond their traditional applications and contribute to addressing goals no. 1 and 2 of the United Nation's sustainable development goals [40,41] for developing countries like Nigeria.

## 2 Study sites

**Fig. 2:**
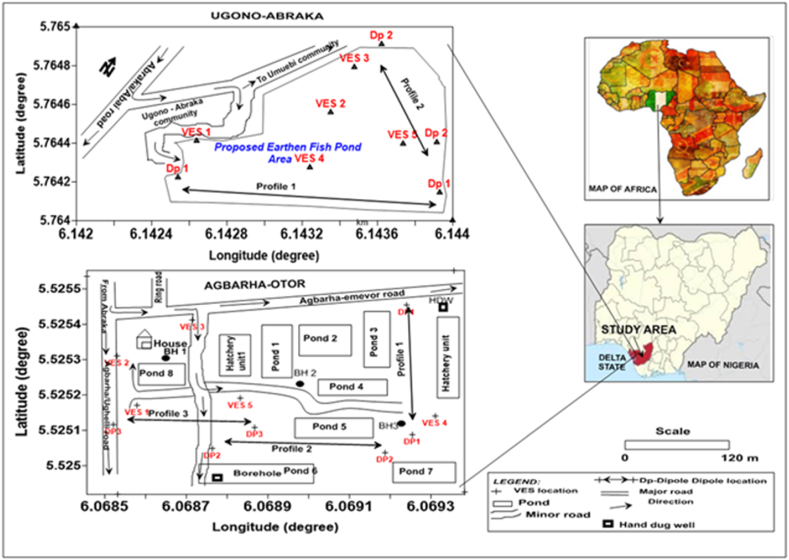
Study sites (Ugono-Abraka in upper left and Agbarha-Otor in the lower left) in Delta State, with their relative position within a map of Nigeria and Africa on the lower and upper right, respectively.

**Fig. 3:**
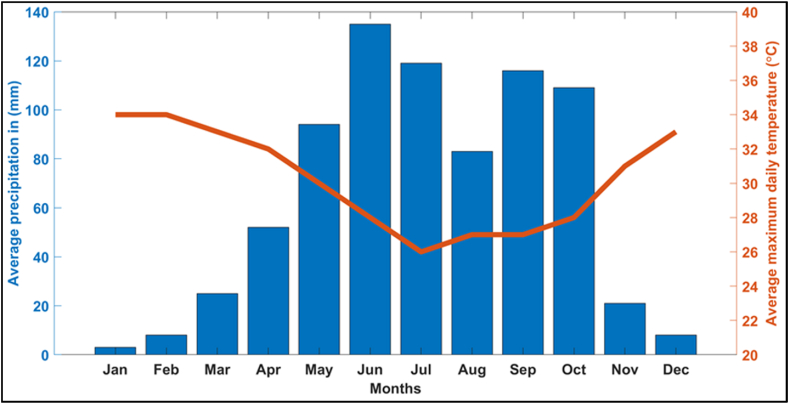
Climate data at Abraka showing the average monthly precipitation as a bar chart (left y-axis) and mean maximum daily temperature as a line graph (right y-axis) over twelve months. The average monthly precipitation was based on measured daily data accumulated over a month. Climate data were modeled using historical data from 1985 and validated with measurements since 2014 [Figure created with data from Meteoblue website, 2022].

This study was conducted at a proposed fishpond site at Ugono-Abraka (Site-1) and an existing fishpond site at Agbarha-Otor (Site-2) communities in the Niger-Delta Region in Southern Nigeria. Site-1 is located between latitude 5.764–5.765° and longitude 6.142–6.144°, while Site-2 is located between latitude 5.5249–5.5255° and longitude 6.0685–6.0693° (Fig. 2). The area is part of the mangrove and freshwater swamp along Nigeria's southern coastline (Fig. 2) [42]. The study areas lie within a Tropical monsoon climate region with an average daily temperature above 25° Celsius and varying average precipitation rates (Fig. 3). High precipitation with a monthly average > 60 mm is observed between May and October and classified as a wet season, while precipitation <60 mm between November and April is classified as a dry season (Fig. 3). While an estimate of varying evapotranspiration is not available due to a data gap, these seasonal climate variables are expected to influence the shallow groundwater level in the region [27]. As a part of the oil-rich Niger-Delta Basin, the regional geology of the area has been extensively studied. It consists mainly of Benin, Agbada, and Akata formations in a top-to-bottom sequence [[43], [44], [45]]. The western and southern segments of the area around the proposed fishpond site at Ugono-Abraka consist of younger Holocene deposits of the Sombreiro-Warri Deltaic Plain [46,47]. These deposits are recent expressions of and a continuation of the Benin Formation [48,49].

The topsoil in the study area, mostly at the Ugono-Abraka site, is mainly hydric soils consisting of a mixture of clay, silt, and sand, which creates seasonally inundated wetlands [50]. The aquifer systems within the study locations include the Sombreiro-Warri Deltaic plain within the Benin Formation, consisting mainly of medium to coarse-grained sands inter-bedded with varying clay contents [51,52]. The clay strata, when laterally extensive, serve as a locally confining unit for the aquifer system, limiting the vertical flow of groundwater.

## 3 Materials and methods

In this study, we used one-dimensional (1D) and two-dimensional (2D) distributions of electrical resistivity and induced polarization with soil cores to estimate the stratigraphic variations at the study sites. Electrical resistivity involves the injection of electrical current (I) using a pair of source electrodes and measuring the resulting potential difference (V) using other electrode pairs, while the resistivity (ρ) is estimated using Ohm's law and accounting for the electrode arrangement [37,53]. Induced polarization involves measuring the reversible charges stored in a porous medium using similar electrode pairs used in electrical resistivity [37]. The ease of acquiring electrical resistivity and induced polarization simultaneously using the same equipment makes their complimentary use quite popular. The theoretical details of both techniques, which have been widely used, are discussed in Binley and Slater [37,54]. For this study, we first acquired 1D electrical resistivity and induced polarization data via electrical sounding to determine bulk resistivity and chargeability distributions at the study sites. We then used the results of bulk electrical resistivity and chargeability estimates to guide the field acquisition of multiple profiles of 2D electrical resistivity and soil cores. Measurement of chargeability was done in the time domain by integrating the decay time of measured voltage after the current injection was stopped. Hence, its unit is reported as a time unit in milliseconds [37]. We further relied on an established petrophysical model from the literature to estimate the soil infiltration coefficients [[55], [56], [57]] within the confines of measured clay content at our study site. Measurements of groundwater levels at existing water wells at the test sites were used to estimate the groundwater flow direction. Knowledge of stratigraphy and estimated hydraulic properties were used to assess the suitability of the sites for establishing an earthen fishpond. Details on each of the methods are discussed in the subsections below.

### 3.1 Field electrical resistivity and induced polarization measurements

Field measurements of electrical resistivity and induced polarization were conducted using an ABEM Terrameter SAS 1000. A total of 10 vertical electrical soundings (VES) stations, with 5 at each test site, were acquired using the Schlumberger and dipole-dipole electrode arrays. Using a minimum AB/2-unit electrode spacing of 1 m with profile lengths ranging between 60 m and 120 m, 12 to 23 electrical soundings were acquired at each VES station. To minimize data error, a current injection of 1.00 mA was done with a minimum and maximum injection cycle of 3 and 6 following standards described in Ref. [53]. We used the reversed quadrupole measurement to assess the accuracy and rejected measurements with over 10% deviation. Both apparent resistivity and chargeability measurements were made at each measurement location. While apparent resistivity was calculated from the injected current, measured potential, and an appropriate electrode configuration factor, the induced polarization (IP) was estimated from the decay function of the voltage over time after turning off the injected current.

Also, 5 transects of 2D electrical resistivity and chargeability were acquired using the same system and setup used for the electrical sounding data acquisition at the two study sites. Two transects were acquired at the Ugono-Abraka site and three at the Agbarha-Otor site (Fig. 2). Due to the lack of appropriate accessories for automated resistivity measurement typical in Nigeria and most developing countries, we designed a 2D data acquisition scheme following guidelines in Everette (2013) [54]. We acquired the data manually using an electrode separation index of n = 1, 2, 3, and 4. The 1D resistivity data obtained were plotted, and the curve matched with multiple iterations using the WinResist computer software. In contrast, the 2D resistivity and IP data were inverted using the Diprowin and IPi2win software. Each inversion's root mean squared error (RMSE) was used to access the model performance.

### 3.2 Soil properties investigation

Soil samples were collected to about 2.5 m depth at the proposed and existing fishpond areas for ground truthing the obtained geophysical models, which were later used to estimate the clay content and stratigraphic variation at the study sites. Static water levels were measured in surrounding boreholes and hand-dug wells (where available) with an electronic water level meter and a handheld Global positioning system (GPS) for each well location. Analysis of the measured water level was used to obtain an approximate groundwater flow direction map.

We measured the clay content in the soil from the collected field soil samples using a sedimentation test. We used the clay content to estimate the soil infiltration coefficients (equation (1)), relying on an established petrophysical relationship relating clay content to infiltration coefficients [55,56]. A theoretical relationship between soil electrical resistivity and salinity for different clay contents were calculated by Ref. [55] using an algorithm developed by Ref. [58] in Shevnin et al. (2006) [56]. This algorithm also calculates soil porosity as a function of clay content in sand-clay soil [56]. Using estimates of clay contents, Slater and Lesmes (2002) [59], Shevnin et al. (2006) [56], and Shevnin et al. (2007) [55] have shown several approximation models for calculating the infiltration coefficient. In this study, we used the Ogilvi approximation model [56] to calculate the infiltration coefficient kf for the unconsolidated sediments at our study sites. The Infiltration coefficient k_f_ is given as:

(1) $$k_{f}={{1.5x10}^{-4}c}^{-2.5}$$

where c is the clay content estimated from electrical resistivity data using the theoretical model in Shevnin et al. (2006) [56].

## 4 Results

### 4.1 Electrical resistivity and induced polarization

#### 4.1.1 Site 1 – Ugono-Abraka

**Fig. 4:**
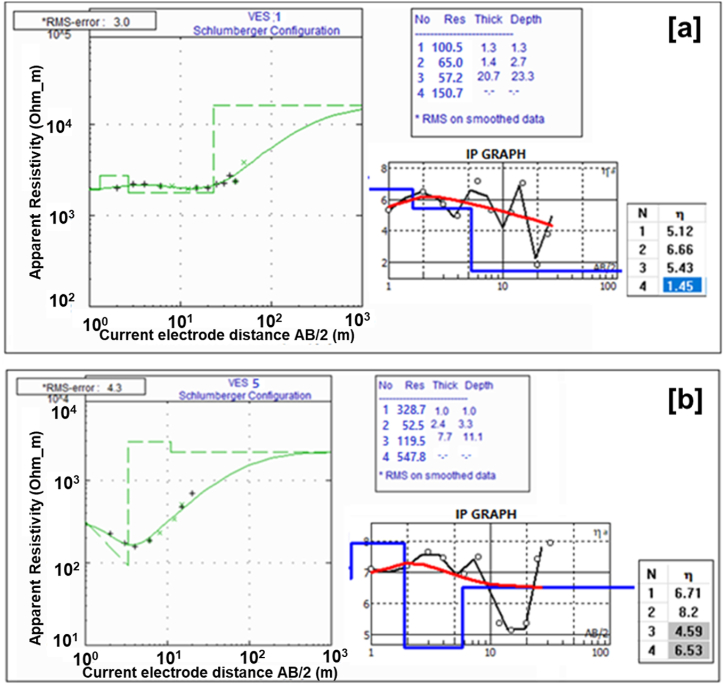
Selected 1D vertical electrical sounding and induced polarization models from station 1 (a) and station 5 (b) at the Ugono-Abraka site (Site-1).

Selected 1D models of electrical resistivity and induced polarization (IP) for the first study site (Site-1 at Ugono-Abraka area) are shown in Fig. 4a and b, while a summary of all VES data is presented in Table A-1 in the appendix section. The 1D models converged with RMSE ranging between 3 and 5%, while all 2D ERT and IP models have RMSE of less than 1%. Generally, the electrical sounding results show a 4-layer resistivity model with a decrease in resistivity with depth apart from the top layer, which has the highest resistivity with a corresponding lowest IP value. The resistivity of the first layer ranges from 100 to 328.7 Ωm, its chargeability ranges from 4.9 to 7.1 mSec, and the thickness range from 0.4 to 1.3 m. The second layer's resistivity range from 32.4 to 96.2 Ωm, its chargeability varies from 4.9 to 11.2 mSec, while the thickness ranges from 0.4 to 2.4 m. The resistivity of the third layer increases compared to the second layer ranging from 57.2 to 120 Ωm. Its chargeability is also relatively higher, ranging from 4.6 to 8.7 mSec, while the estimated thickness shows a large variation ranging from 0.7 m to 20.4 m. The resistivity of the fourth layer ranges from 150.7 Ωm to 547.8 Ωm.

**Table 1:** Soil properties estimation based on clay content values found from VES resistivity at Ugono – Abraka.

Location	Number Layer	Resistivity ρ (Ωm)	Chargeability m_a_ (msecs)	Clay content (%)	K_f_ ×10^−5^ (m/sec)	K_f_ (m/day)	Transmissivity (m^2^/day)
VES 1	1	100.5	5.12	3	1.8	1.536	1.997
	2	65	6.66	5	0.6	0.477	0.667
	3	57.2	5.43	7	0.3	0.221	4.567
	4	150.7	1.45	-.-	-.-	-.-	-.-
VES 2	1	120.9	7.57	2.5	2.7	2.332	0.933
	2	96.2	8.35	3.4	1.3	1.153	1.845
	3	57.4	8.73	5	0.6	0.477	2.145
	4	240.6	9.52	-.-	-.-	-.-	-.-
VES 3	1	128.4	4.88	2.4	3.0	2.560	2.560
	2	66.2	11.2	5.1	0.5	0.456	0.182
	3	96.3	6.4	3.4	1.3	1.153	0.807
	4	377.4	9.76	-.-	-.-	-.-	-.-
VES 4	1	173.2	7.11	1.8	5.7	4.947	4.453
	2	32.4	4.92	9.8	0.1	0.102	0.082
	3	120	7.16	2.5	2.7	2.332	12.357
	4	534.5	6.13	-.-	-.-	-.-	-.-
VES 5	1	328.7	6.71	1.3	12.1	10.423	10.423
	2	52.5	8.2	5.6	0.4	0.368	0.883
	3	119.5	4.59	2.9	1.9	1.660	12.780
	4	547.8	6.53	-.-	-.-	-.-	-.-

**Fig. 5:**
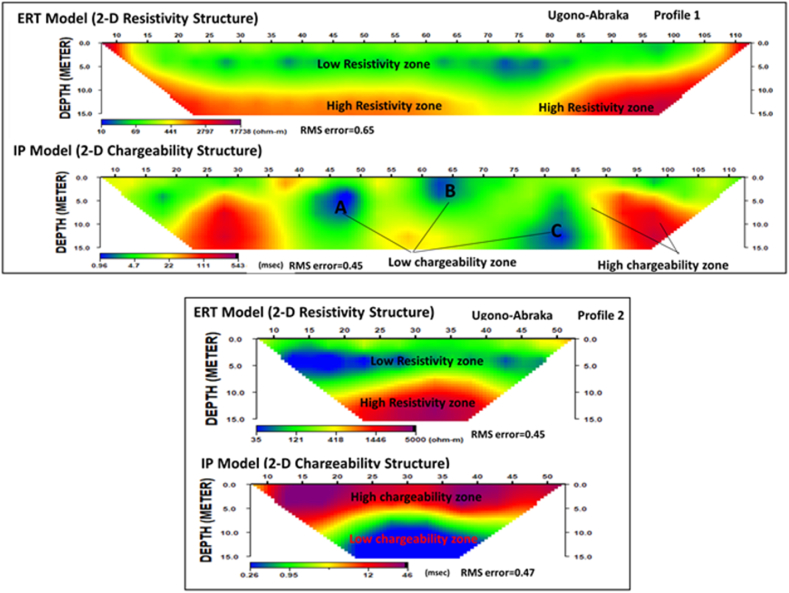
2D distribution of electrical resistivity and induced polarization from Profiles 1(top 2) and 2 (bottom 2) at Ugono-Abraka.

The 2D electrical resistivity and IP profiles (Fig. 5 - profiles 1, 2) show similar results with a 4-layer resistivity model, while the IP show 3 major zones. The 2D models capture both resistivity and IP heterogeneities within the subsurface. Higher resistivities are present in the top layer of profiles 1 and 2 (Fig. 5), with resistivity ranging from 80 to 450 Ωm for layer 1. This is underlain by a low resistivity layer 2 with resistivities less than 90 Ωm. It is worth noting that the low resistivity zone in layer 2 (<90 Ωm) shows discontinuities with high resistivities (about 180 Ωm) in between the low resistivities. Layer 2 is underlain by layers 3 and 4 with increasing resistivities like the VES results (Fig. 5). The IP models show contrasting results for profiles 1 and 2. Profile 1 of Fig. 5 shows discontinuous zones of low (<20 mSec) and high (20–180 mSec) chargeability while profile 2 of Fig. 5 shows 3 different chargeability zones in a layered format with the IP decreasing with depth. Layer 1 has discontinuous and higher IP values (>20 mSec), while layer 2 has IP values between 10 and 20 mSec. The third layer at a depth below 5 m is characterized by low IP of less than 10 mSec.

#### 4.1.2 Site 2 – Agbarha-Otor

**Fig. 6:**
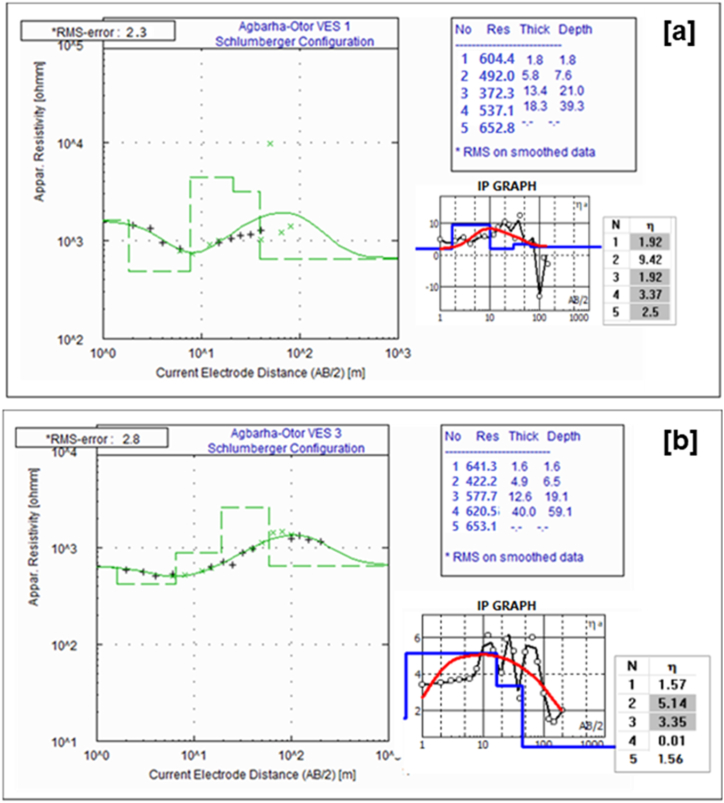
Selected 1D models of vertical electrical sounding and induced polarization from station 1 (a) and station 3 (b) at the Agbarha-Otor site (Site-2).

The 1D electrical sounding sections for study site 2 (Agbarha-Otor area) show a 4- to 5-layer resistivity model with RMSE ranging between 2 and 3%. Selected electrical sounding results showing 1D electrical resistivity and IP are presented in Fig. 6 a, b, while a summary of the resistivity, chargeability, and depth for all VES stations are presented in Table A-2 in the Appendix section. Generally, the resistivity at the site increases with depth besides the top layer (layer 1), which has a higher resistivity (Fig. 6 a, b). The first layer's resistivity ranges from 251 to 642 Ωm, its chargeability ranges from 1.6 to 8.3 mSec, and the thickness ranges from 1.0 to 2.5 m. The second layer's resistivity ranges from 411.3 to 459.8 Ωm, its chargeability ranges from 1.3 to 9.5 mSec, and the thickness ranges from 2.3 to 5.8 m. The third layer's resistivity ranges from 266.3 to 710.3 Ωm, its chargeability ranges from 1.9 to 13.1 mSec, and the thickness ranges from 3.6 to 16.7 m. The resistivity of the fourth layer ranges from 479.4 to 826.4 Ωm, its chargeability ranges from 0.1 to 7.8 mSec, and the thickness ranges from 4.3 to 40.0 m. The fifth layer has the highest resistivity values ranging from 635.7 to 1200.5 Ωm with an undetermined thickness.

**Table 2:** Soil properties estimation based on clay content values found from VES at Agbarha – Otor.

**Location**	**Number Layer**	**Resistivity** **ρ (Ωm)**	**Chargeability m**_**a**_**(msecs)**	**Clay content (%)**	**K**_**f**_**x10**^**−**^**^4^ (m/sec)**	**K**_**f**_**(m/day)**	**Transmissivity (m**^**2**^**/day)**
VES 1	1	604.4	1.92	0.9	2.800	24.195	43.551
	2	492	9.42	1.15	1.597	13.802	80.051
	3	372.3	1.92	1.4	1.18	8.796	117.871
	4	537.1	3.37	-.-	-.-	-.-	-.-
	5	652.8	2.5	-.-	-.-	-.-	-.-
VES 2	1	333	7.69	1.4	1.018	8.796	21.991
	2	459.8	1.25	1.2	1.449	12.520	48.829
	3	710.3	13.1	0.8	3.667	31.685	529.148
	4	816.5	7.81	-.-	-.-	-.-	-.-
	5	913.3	5.52	-.-	-.-	-.-	-.-
VES 3	1	641.3	1.57	0.9	2.800	24.195	38.712
	2	422.2	5.14	1.2	1.449	12.520	61.349
	3	577.7	3.35	1	2.200	19.008	239.501
	4	620.5	0.01	-.-	-.-	-.-	-.-
	5	653.1	1.56	-.-	-.-	-.-	-.-
VES 4	1	310	3.38	1.4	1.018	8.796	10.556
	2	431.5	3.28	1.2	1.449	12.520	30.048
	3	266.3	5.32	1.6	0.750	6.479	34.986
	4	828.4	7.16	-.-	-.-	-.-	-.-
	5	1200.5	3.08	-.-	-.-	-.-	-.-
VES 5	1	298.3	6.3	1.55	0.806	6.967	6.271
	2	411.3	3.78	1.2	1.449	12.520	28.796
	3	286.5	7.53	1.52	0.843	7.286	26.231
	4	479.4	3.91	-.-	-.-	-.-	-.-
	5	635.7	0.83	-.-	-.-	-.-	-.-

**Fig. 7:**
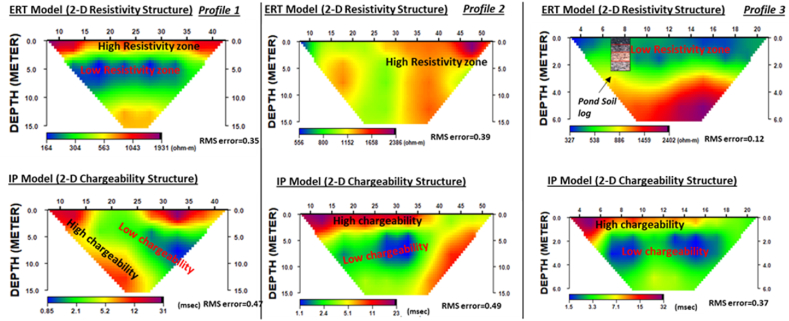
2D distribution of electrical resistivity and induced polarization from Profiles 1 (left), 2 (middle), and 3 (right) at Agbarha-Otor.

Results of all 3 transects’ 2D electrical resistivity and IP are presented in Fig. 7 (Profiles 1–3), with all models having RMSE of less than 1%. High variations in resistivity and chargeability are observed, with profile 1 showing a top layer with high resistivities ranging from 430 to 1, 200 Ωm, extending to a maximum depth of 3 m. A low resistivity zone with values ranging from 175 to 250 Ωm is also observed running across the transects at a depth of 3–9 m. Profile 2, in contrast to 1, shows lateral changes in resistivity with increasing values from 550 Ωm on the left to 2400 Ωm on the right of the profile. Profile 3 has resistivity increasing downwards with relatively lower resistivities ranging from 330 to 550 Ωm at the top, extending to about 3 m. This is underlain by a higher resistivity unit with resistivity ranging from 550 to 1400 Ωm. Like the resistivity, the IP results for profiles 1 to 3 also show significant variability, with that profile 1 showing a dipping anomaly trend in the chargeability with values decreasing from 4 to 1 mSec. High chargeability values ranging from 5 to 20 mSec are observed at the left- and right-hand side of the profile (Fig. 7). The IP signals in profiles 2 and 3 are similar, with high chargeability values at the top 2 m ranging from 8 to 13 mSec and a low chargeability zone at depths of 3–10 m with values less than 3 mSec.

### 4.2 Soil properties

A summary of the estimated soil properties for both study sites, i.e., Ugono-Abraka and Agbarha-Otor, are presented in Table 1, Table 2 The clay content in Ugono-Abraka ranges from 1.1 to 9.8%, with an estimated infiltration coefficient k_f_ ranging from 0.05 to 9.5 m/day. For the study site at Agbarha-Otor, the clay content ranges from 0.3 to 1.6%, while the estimated infiltration coefficient k_f_ ranges from 4.6 to 304.3 m/day.

**Fig. 8:**
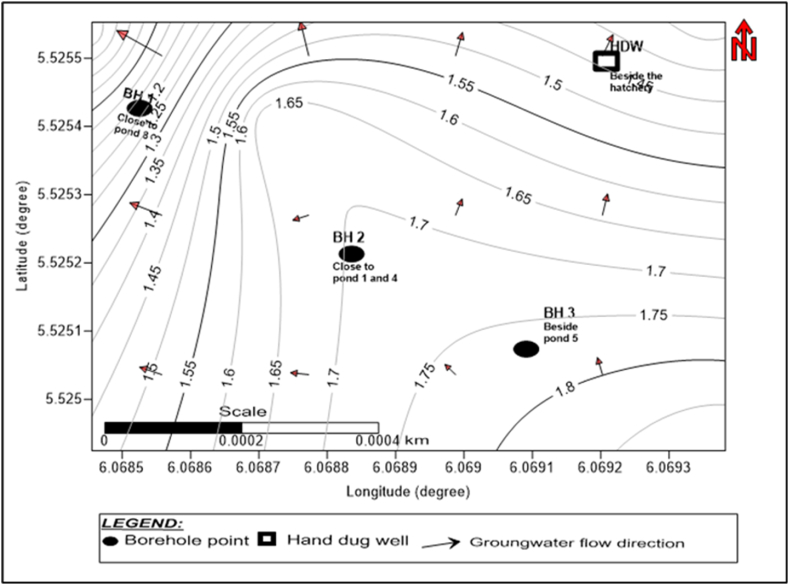
Contour map showing the distribution of static hydraulic heads (water levels) at the Ugono-Abraka site. Water levels were measured at boreholes and hand-dug wells in the surrounding area.

The static water levels measured from existing boreholes at study site 2 (Agbarha-Otor) were used to construct the relative groundwater flow direction shown in Fig. 8, with water levels ranging from 0.8 to 1.9 m (Appendix A-3). The groundwater flows eastwards toward a pond serving as a sink.

## 5 Discussion

### 5.1 Subsurface geoelectrical distribution at study site-1 (Ugono – Abraka)

**Fig. 9:**
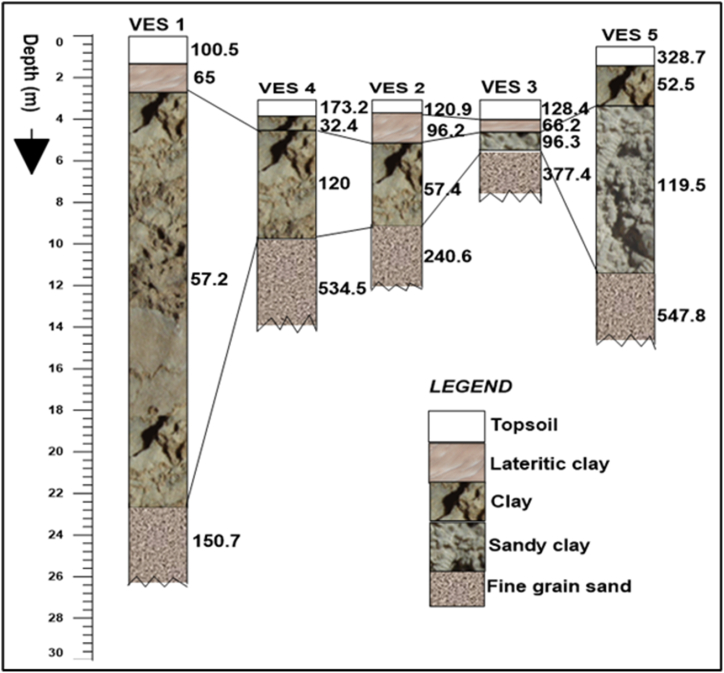
Interpreted geoelectric sections of VES 1 to VES 5 of Ugono – Abraka.

**Fig. 10:**
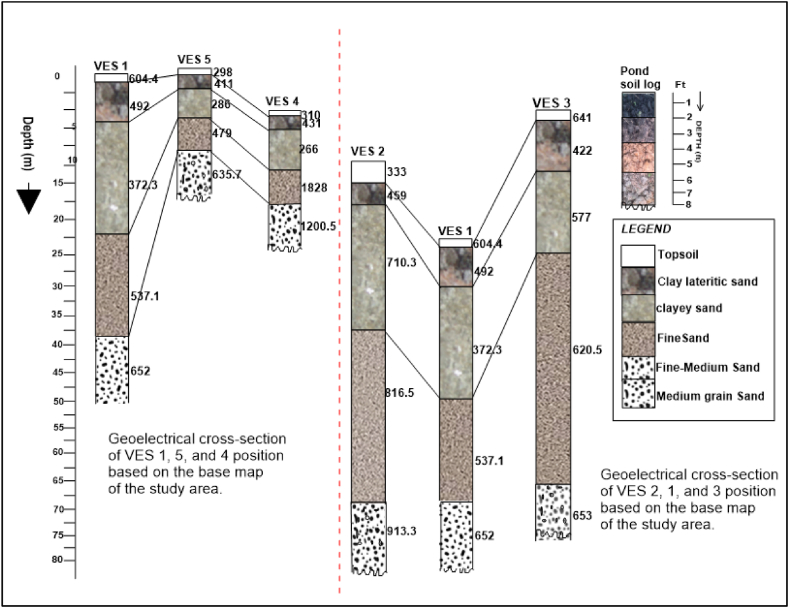
Interpreted geoelectric sections VES 1 to VES 5 of Agbarha-Otor.

Electrical-sounding data obtained in this study were used to create a geoelectrical section with interpretations showing the possible corresponding lithology using resistivity values (Fig. 9, Fig. 10). The lithology generally controls groundwater seepage rates as clay-rich zones typically have low seepages and should retain water for a longer time, thus much more suitable for establishing earthen fishponds [56,[59], [60], [61]]. The interpretations follow the established relationship between resistivity and sediment types [[57], [58], [59]]. Based on the resistivity and chargeability distributions from both 1D and 2D models (Fig. 4, Fig. 5, Fig. 6, Fig. 7) validated with sediments collected from test pits and exposed walls of nearby fishponds (Appendix A-4), the shallow subsurface stratigraphy at the study site 1 (i.e., Ugono-Abraka) is interpreted to consists of loamy topsoil with high organic matter content, clay-rich lateritic sediments, sandy clay, and fine to medium grain sands from a top to bottom sequence (Fig. 9). This interpretation is similar to earlier work delineating the subsurface stratigraphy close to the site [50]. The geoelectric sections from electrical soundings 1, 4, and 5 show thicker topsoil sequences. These are mainly hydric soils with varying clay contents in the southern segment of the study site with greater depth to the fine to medium-grained sand (Fig. 9). This segment would be more suitable for establishing an earthen fishpond as water retention would be higher [27,56] than the sand-rich segment.

The 2D IP results at site 1 (Fig. 5, profiles 1 and 2) complement our interpretation of the 4-layered model VES and 2D resistivity results. Particularly in profile 2 of Fig. 5, the low electrical resistivity zones (75–120 Ωm) correspond to the high IP zone (10–24 mSec). The relatively high chargeability confirms our interpretation of a clay-rich zone. This highlights the complimentary advantage of measuring IP signals simultaneously with resistivity as it could be used to differentiate low resistivity signatures resulting from fluid content and that from clay. While resistivity is sensitive to both the fluid and clay content, the IP signals are only sensitive to the clay content [37]. A similar complementary trend is observed at the lower section of the resistivity and IP models (Fig. 5), where a high resistivity zone corresponds to a low chargeability confirming our interpretation of a sandy unit. In contrast to the direct opposite correlation between resistivity and IP in Profile 2 of Fig. 5, such a correlation is unclear in Profile 1. We interpret this to result from variation in the clay/sand content. The low resistivity zones with corresponding low chargeability may result from low clay content assuming less measurement error.

### 5.2 Subsurface geoelectrical distribution at study site-2 (Agbarha-Otor)

**Fig. 11:**
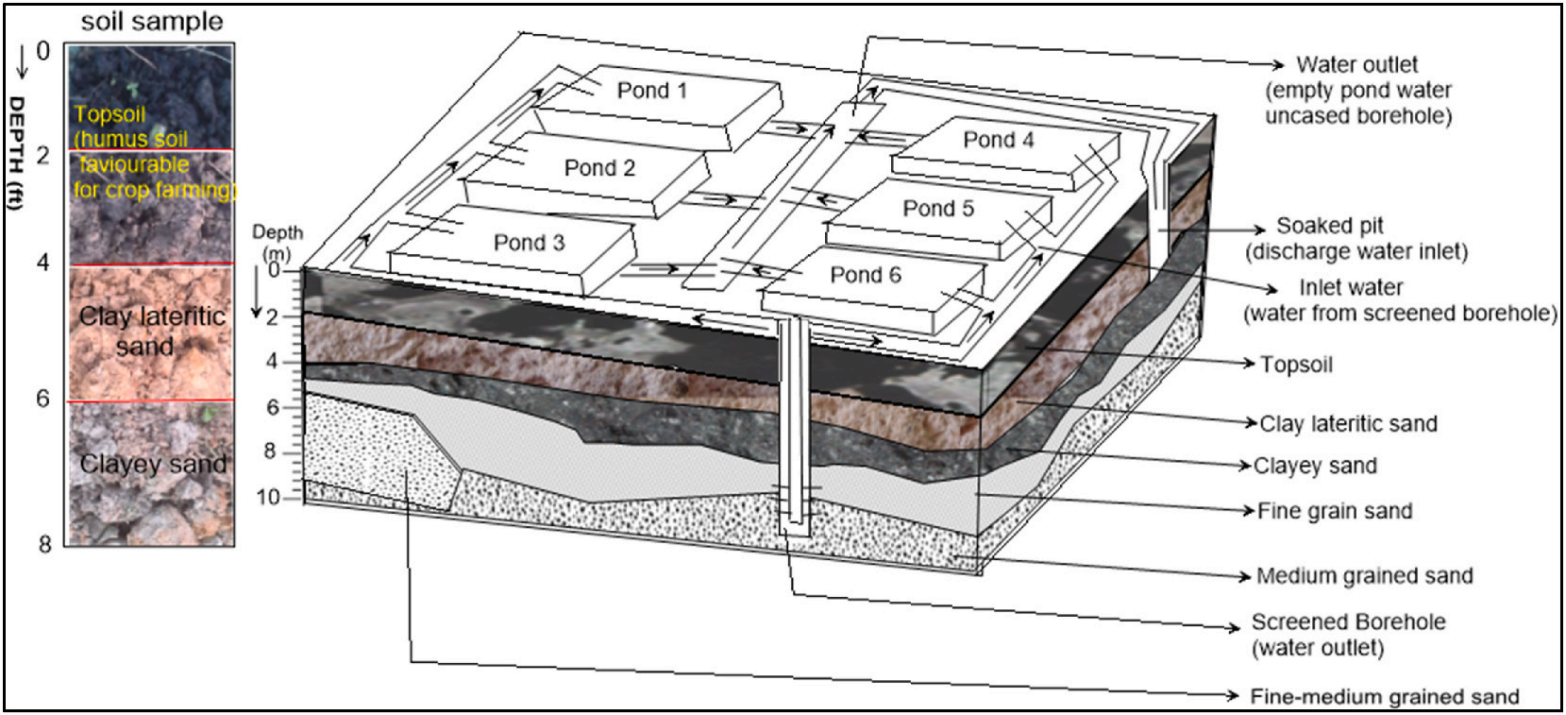
Soil sample collected (left) and Conceptual mode (right) of the research site.

Similar to the interpretation of site 1, we used results of the electrical resistivity and IP distributions to infer the shallow subsurface stratigraphy at site 2 (Agbarha-Otor). The 4- to 5-layer model in VES 1 to 5 (Fig. 6 a, b and Table 2) is interpreted as a topsoil, clayey lateritic sand, clayey sand, fine grain sand, and fine to medium grain sand in a top to bottom sequence (Fig. 10). The IP result also provides complementary information for interpreting the 2D resistivity distribution (Fig. 7). Like what was observed in resistivity profiles from site 1, the low resistivity zones in profiles 1 and 3 correspond to relatively higher chargeability which are interpreted as clay-rich zones (Fig. 7). At study site 2, the interpreted sandy layer is somewhat deeper and is saturated with a depth ranging from 6.8 to 23.1 m. This corresponds to water level measurement in the areas with static hydraulic heads varying between 0.81 and 1.90 m, as shown in Appendix A-3. Strong IP effects were also observed but with high resistivity potentially due to the significant influence of the surface material grain sizes, shape of particles, mineral conductivity, and the grain packing on chargeability [62,63]. Therefore, this layer can also retain water due to its significant clay content. By combining results of resistivity and IP distribution, lithological data from soil samples, and observed sediment types exposed at the pond walls, we developed a 3D conceptual model showing the subsurface architecture for the existing fishpond at study site 2 (Fig. 11).

### 5.3 Implication of subsurface condition on earthen fishpond at the study sites

Based on the soil clay contents, we estimated the infiltration coefficient shown in Table 1, Table 2 While these estimates used existing petrophysical relationships [56], which are inherently non-unique [42], they provide a first-level approximation for a quantitative assessment of the hydraulic conditions of the site where in-situ or laboratory measurements are not available. The clay content for the site at Ugono-Abraka (1.1–9.8%) is significantly higher than that at Agbarha-Otor (0.3–1.6%). The infiltration coefficient, as expected, shows an opposite trend with lower values at Ugono-Abraka (0.05–9.5 m/day) compared to Agbarha-Otor (4.6–304.3 m/day). These results indicate that the subsurface conditions in the Ugono-Abraka area are within the range of very slow to slow seepages, while those within the Agbarha-Otor area are within the range of slow to moderate seepages using the FAO standards for seepage in soil based on infiltration coefficient 56]. Hence, the results reveal that the Ugono-Abraka site, where a mid-sized earthen fishpond is proposed, can provide adequate water holding capacity throughout the year for the fishpond [27]. However, the higher infiltration rates at the Agbarha-Otor site will make the soil incapable of retaining water year-round [27]. This explains the current experience of fish farmers complaining of their fishponds losing water significantly and drying up during the dry season (oral communication with fish farmers at Agbarha-Otor). Given the relatively low precipitation (<60 mm) and high average daily temperatures (>30° Celcius) (see Fig. 2), water loss will be higher at the Agbarha-Otor site, where this is via both vertical flow and evapotranspiration. The obtained estimates of soil hydraulic properties in this study correspond well with that of earlier studies by Iserhien-Emekeme et al. [60], with reported average k_f_ value of 38.88 m/day and Aweto and Akpoborie [61] with k_f_ values ranging between 10.5 and 45.7 m/day.

Estimates of the distribution of the soil properties obtained in this study can inform the design of the earthen fishponds [27]. The presence of soil with potentially low clay content and high infiltration coefficients at the Agbarha-Otor site (Site-2) does not preclude the site from being used for earthen fishpond purposes. However, obtaining this information via prior hydrogeophysical measurements could be used to improve the design of the fishpond to prevent water seepages. A typical approach for doing this involves sealing the base of the pond with clayey soils with low infiltration rates to prevent potential water loss [27]. In such instances, water could be added to the pond during the dry season when the evapotranspiration rate is high without dealing with vertical water loss via seepages.

## 6 Conclusions

In this study, we showed the innovative use of non-invasive geophysical methods, including electrical resistivity and induced polarization to assess sites’ suitability for establishing earthen fishponds in the Niger Delta region of Nigeria. ID and 2D models of electrical resistivity and chargeability were combined with limited lithological data to delineate the shallow subsurface stratigraphy. We show the complimentary use of induced polarization with electrical resistivity for differentiating low resistivity clay zones from fluid content effect relying on the sensitivity of induced polarization, unlike resistivity mostly to the soil texture and less to the fluid content.

We also showed the value of petrophysical relationships in characterizing sites with no prior hydraulic data by using existing petrophysical relationships to estimate the infiltration rates based on measured clay content. While such petrophysical relationships have inherent limitations, including their site-specific application, and should be used cautiously, they offer an approach for obtaining quantitative information on the site's hydraulic properties for this study. We calculated the infiltration coefficients based on the clay content to quantify potential percolation and the corresponding water retention at both study sites. Using the geophysical models of electrical resistivity and induced polarization to delineate the spatial extent of the clay layers at the sites provided an opportunity to spatially delineate zones with potentially low infiltration rates suitable for earthen fishponds.

The results of this study revealed that the Ugono-Abraka site, where earthen fishing ponds are proposed, has subsurface conditions that are more suitable for earthen fishponds compared to that of Agbarha-Otor where there is an existing earthen fishing pond. This clay-rich layer at 3 – 5 m depth at the Ugono-Abraka site can restrict the vertical flow of water, allowing more water retention. While the results of this study provide insight into the hydrostratigraphic variability at the study sites, the interpretation is limited due to the lack of sufficient soil cores to adequately ground-truth the geophysical model. Future work is planned to extend the electrical resistivity and induced polarization models to 3D, collect soil samples, perform field hydraulic tests and develop a numerical model integrating the geophysical and hydraulic data to assess water retention capacity within proposed and existing earthen fishponds.

## Author contribution statement

Efemena Destiny Emmanuel; Edmund A. Atakpo: Conceived and designed the experiments; Performed the experiments; Analyzed and interpreted the data; Contributed reagents, materials, analysis tools or data; Wrote the paper.

Kennedy O. Doro: Performed the experiments; Analyzed and interpreted the data; Contributed reagents, materials, analysis tools or data; Wrote the paper.

Ruth E. Iserhien-Emekeme: Conceived and designed the experiments; Analyzed and interpreted the data; Contributed reagents, materials, analysis tools or data.

## Data availability statement

Data will be made available on request.

## Additional information

No additional information is available for this paper.

## Funding

This research received no external funding.

## Declaration of competing interest

The authors declare that they have no known competing financial interests or personal relationships that could have appeared to influence the work reported in this paper.

## Figures and Tables

**Table:** Summary of Results obtained from Computer iteration Model Parameters at Ugono – Abraka

Location	Number Layer	Resistivity ρ_a_ (Ωm)	Chargeability η_a_ (msecs)	Thickness h (m)	Depth d(m)	Inferred lithology
VES 1	1	100.5	5.12	1.3	1.3	Topsoil
	2	65	6.66	1.4	2.7	Lateritic clay
	3	57.2	5.43	20.7	23.3	Sandy clay
	4	150.7	1.45	-.-	-.-	Fine grain sand
VES 2	1	120.9	7.57	0.4	0.4	Topsoil
	2	96.2	8.35	1.6	2	Lateritic clay
	3	57.4	8.73	4.5	6.4	Sandy clay
	4	240.6	9.52	-.-	-.-	Fine grain sand
VES 3	1	128.4	4.88	1	1	Topsoil
	2	66.2	11.2	0.4	1.4	Lateritic clay
	3	96.3	6.4	0.7	2.1	Sandy clay
	4	377.4	9.76	-.-	-.-	Fine grain sand
VES 4	1	173.2	7.11	0.9	0.9	Topsoil
	2	32.4	4.92	0.8	1.7	clay
	3	120	7.16	5.3	7	Sandy clay
	4	534.5	6.13	-.-	-.-	Fine grain sand
VES 5	1	328.7	6.71	1	1	Topsoil
	2	52.5	8.2	2.4	3.4	clay
	3	119.5	4.59	7.7	11.1	Sandy clay
	4	547.8	6.53	-.-	-.-	Fine grain sand

**Table:** Summary of Results obtained from Computer iteration Model Parameters at Agbarha – Otor.

Location	Number Layer	Resistivity ρ_a_ (Ωm)	Chargeability η_a_ (msecs)	Thickness h (m)	Depth d(m)	Inferred lithology
VES 1	1	604.4	1.92	1.8	1.8	Topsoil
	2	492	9.42	5.8	7.6	Clayey lateritic sand
	3	372.3	1.92	13.4	21	Clayey sand
	4	537.1	3.37	18.3	39.3	Fine grain sand
	5	652.8	2.5	-.-	-.-	Fine – medium grain sand
VES 2	1	333	7.69	2.5	2.5	Topsoil
	2	459.8	1.25	3.9	6.4	Clayey lateritic sand
	3	710.3	13.1	16.7	23.1	Clayey sand
	4	816.5	7.81	29.9	53	Fine grain sand
	5	913.3	5.52	-.-	-.-	Fine – medium grain sand
VES 3	1	641.3	1.57	1.6	1.6	Topsoil
	2	422.2	5.14	4.9	6.5	Clayey lateritic sand
	3	577.7	3.35	12.6	19.1	Clayey sand
	4	620.5	0.01	40	59.1	Fine grain sand
	5	653.1	1.56	-.-	-.-	Fine – medium grain sand
VES 4	1	310	3.38	1.2	1.2	Topsoil
	2	431.5	3.28	2.4	3.6	Clayey lateritic sand
	3	266.3	5.32	5.4	9	Clayey sand
	4	828.4	7.16	4.3	13.3	Fine grain sand
	5	1200.5	3.08	-.-	-.-	Fine – medium grain sand
VES 5	1	298.3	6.3	0.9	0.9	Topsoil
	2	411.3	3.78	2.3	3.2	Clayey lateritic sand
	3	286.5	7.53	3.6	6.8	Clayey sand
	4	479.4	3.91	6	12.9	Fine grain sand
	5	635.7	0.83	-.-	-.-	Fine – medium grain sand

**Table:** Static water level from borehole/hand dug well measurement at Agbarha-Otor

Borehole/dug well	Location	Latitude Longitude	Ground Surface elevation (m)	Static water level (m)	Well Depth (meter)
BH1	Close to pond 8	05°31^1^33.1^11^ N 006^0^04^1^11.0^11^ E	10	1.7	19.33
BH2	Close to pond 1 and 4	05°31^1^38.7^11^ N 006^0^04^1^04.5^11^ E	14	0.81	28.32
BH3	Beside pond 5	05°31^1^35.7^11^ N 006^0^04^1^03.3^11^ E	25	1.32	23.28
HDW	Beside the hatchery	05°31^1^35.8^11^ N 006^0^04^1^03.4^11^ E	27	1.90	5.81
